# Safety evaluation of afatinib in patients with a history of interstitial lung disease using data from an administrative claims database in Japan

**DOI:** 10.1007/s11096-026-02114-2

**Published:** 2026-03-17

**Authors:** Ryo Inose, Kuniyoshi Hayashi, Toshiyuki Sakaeda

**Affiliations:** 1 Laboratory of Clinical Pharmacoepidemiology, Kyoto Pharmaceutical University, 5 Misasagi-nakauchi-cho, Yamashina-ku, Kyoto, 607-8414 Japan; 2 Faculty of Data Science, Kyoto Women’s University, 35 Kitahiyoshi-cho, Imakumano, Higashiyama-ku, Kyoto, 605-8501 Japan; 3 Laboratory of Pharmacokinetics, Kyoto Pharmaceutical University, 5 Misasagi-Nakauchi-cho, Yamashina-ku, Kyoto, 607-8414 Japan

**Keywords:** Administrative claims database, Afatinib, Classification and regression tree, Interstitial lung disease

## Abstract

**Introduction:**

Afatinib, an epidermal growth factor receptor tyrosine kinase inhibitor (EGFR-TKI), is an effective treatment agent for lung cancer with uncommon epidermal growth factor receptor (EGFR) mutations. EGFR-TKIs should be administered with caution in patients with a history of interstitial lung disease (ILD); however, such patients are often excluded from clinical trials, and evidence on afatinib safety in this population remains limited.

**Aim:**

To exploratorily evaluate the safety profile of afatinib in patients with a history of ILD using an administrative claims database and investigate whether severe ILD events occur uniformly across patient backgrounds or cluster within specific patient profiles.

**Method:**

We analyzed data of patients who received afatinib between May 1, 2014 and November 30, 2020 from the administrative claims database provided by Medical Data Vision Co., Ltd. Severe ILD was defined using two claims-based operational definitions: (1) a broader primary definition combining ILD diagnostic codes and high-dose intravenous corticosteroid administration; (2) a stricter definition requiring intravenous methylprednisolone for sensitivity analysis. Classification and Regression Trees (CART) were used to exploratorily identify patient profiles associated with severe ILD.

**Results:**

Among 2,174 patients treated with afatinib, 12.3% (267/2,174) had a history of ILD. Using the primary definition, severe ILD occurred in 6.0% (16/267) of these patients, whereas 3.4% (9/267) met the stricter definition. Although CART analyses yielded different split variables and cutoff values across the two definitions, both consistently indicated that severe ILD events were concentrated within specific patient profiles rather than being uniformly distributed across the population.

**Conclusion:**

In patients with a history of ILD, the incidence of severe ILD following afatinib treatment was clarified. CART-derived findings should be interpreted as exploratory signals suggesting clustering of severe ILD events within particular patient profiles. However, as severe ILD was identified using claims-based operational definitions, outcome misclassification cannot be excluded despite sensitivity analyses. Further studies are warranted to validate these findings in independent real-world datasets with richer clinical detail and to support more precise stratification of severe ILD occurrence.

**Supplementary Information:**

The online version contains supplementary material available at 10.1007/s11096-026-02114-2.

## Impact statement

- Of the 2,174 patients who received afatinib, 12.3% (267 patients) had a history of interstitial lung disease (ILD). In clinical practice, afatinib may sometimes need to be administered to patients with a history of ILD.
- In patients with a history of ILD, the incidence of severe ILD following afatinib treatment was 6.0%. Careful monitoring is required when afatinib is administered to such patients.
- In exploratory analyses, severe ILD events appeared to occur more frequently in younger patients with a history of ILD. These findings should be interpreted cautiously and may support closer clinical assessment and monitoring, rather than serving as definitive criteria for treatment selection.

## Introduction

Lung cancer is the leading cause of cancer-related deaths worldwide [1]. Cytotoxic and molecular targeted anticancer drugs are frequently used in lung cancer treatment. In particular, epidermal growth factor receptor tyrosine kinase inhibitors (EGFR-TKIs) represent the mainstay of treatment for patients with lung cancer harboring mutations in the epidermal growth factor receptor (*EGFR*) gene [2, 3].

Various types of EGFR-TKIs, such as gefitinib, afatinib, and osimertinib, are available for patients with lung cancer. However, reports on the therapeutic effects of EGFR-TKIs in patients with uncommon *EGFR* mutations are scant. Recently, afatinib has garnered attention as an effective treatment agent in patients with uncommon *EGFR* mutations [4, 5]; it has the potential to become one of the first-line drugs for patients with uncommon *EGFR* mutations [5].

EGFR-TKIs often cause well-recognized adverse events, such as diarrhea and rashes [6, 7]. In addition, various aspects of their safety profiles of afatinib have been evaluated in clinical trials [8–10] and post-marketing studies [11, 12]. Drug-induced interstitial lung disease (ILD) is a rare but potentially fatal event that requires careful attention. Therefore, EGFR-TKIs should be administered with caution in patients with a history of ILD. However, as EGFR-TKIs are key drugs for patients with lung cancer harboring *EGFR* mutations [2, 3], they are sometimes administered to patients with a history of ILD in clinical settings [13, 14].

In a multicenter study involving 58 patients with a history of EGFR-TKI–induced ILD who were re-administered EGFR-TKIs, grade 3 or higher ILD occurred in 6.9% of patients [15]. However, the small cohort size limited external validity, and the potential association between patient characteristics and severe ILD incidence, as well as the distribution and patterns of severe ILD in this population, were not fully elucidated. Moreover, as patients with a history of ILD are often excluded from clinical trials [9, 10], evidence regarding the safety of afatinib in this population remains limited. Real-world data (RWD) can complement trial evidence by enabling safety evaluations in broader patient populations and facilitating exploration of whether severe ILD events occur uniformly across patient backgrounds or are concentrated within specific clinical profiles. However, such analyses for afatinib in patients with a history of ILD have not been sufficiently reported.

## Aim

This exploratory study aimed to evaluate the safety profile of afatinib in patients with a history of ILD using an administrative claims database as a form of RWD, and investigate whether severe ILD events are uniformly distributed across patient backgrounds or concentrated in specific patient subgroups.

## Methods

### Database and patient selection

We used data from an administrative claims database provided by Medical Data Vision Co., Ltd. (MDV; Tokyo, Japan). The MDV database is a real-world data source derived from routine clinical practice and has been widely used in epidemiological and pharmacoepidemiological research in Japan [16, 17]. It contains longitudinal information on diagnoses, procedures, and prescription records from hospitals operating under the Diagnosis Procedure Combination (DPC) system and covers approximately 30% of DPC hospitals nationwide. Although detailed clinical information (e.g., laboratory results and imaging findings) is not available, this database is suitable for large-scale observational studies evaluating treatment patterns and safety outcomes. Our study involved patients who were administered afatinib for the first time for lung cancer treatment between May 1, 2014 and November 30, 2020. The index date was defined as the date of first afatinib administration. For patients administered multiple periods of afatinib treatment, data from only the initial treatment period were included. The exclusion criteria were as follows: (1) patients with missing background information; (2) patients with unavailable patient characteristic data for the year prior to afatinib treatment; and (3) patients without a history of ILD. The study cohort was identified through a stepwise selection process, with the final analytic population restricted to patients with a documented history of ILD prior to afatinib initiation.

### Drug and diagnosis definitions

We defined the drugs according to the European Pharmaceutical Marketing Research Association Anatomical Therapeutic Chemical classification (Online Resource 1) and disease names according to the International Classification of Diseases, 10th edition Online Resource 2).

We retrospectively investigated the data on drug administration and medical history for up to 1 year before the first administration of afatinib. The DPC data used in this study did not include a disease name indicating severe ILD. Therefore, severe ILD was operationally defined using diagnosis and treatment patterns recorded in claims data. A broader definition was applied in the primary analysis to increase sensitivity for detecting clinically relevant severe ILD treated in routine practice. In contrast, a stricter definition was used in sensitivity analysis to improve outcome specificity. To support transparency and reproducibility, the operational definitions used in the primary and sensitivity analyses are summarized in Online Resource 3.

In clinical practice, moderate to severe ILD is generally treated with high-dose corticosteroids, such as methylprednisolone (500–1000 mg/day) or prednisolone (0.5–1.0 mg/kg/day) [18]. However, clinicians may adjust or reduce these doses depending on patient condition, including age, comorbidities, and infection risk. Therefore, we defined high-dose steroids as intravenous methylprednisolone ≥ 500 mg/day and intravenous prednisolone ≥ 10 mg/day, considering variations in actual clinical practice. For the primary analysis, severe ILD was defined as a diagnosis of ILD and the administration of high-dose steroids (intravenous methylprednisolone ≥ 500 mg/day and intravenous prednisolone ≥ 10 mg/day) within the period from the first afatinib administration to 1 month after the final administration.

To assess robustness and potential outcome misclassification, a sensitivity analysis was conducted using a stricter definition of severe ILD. In this sensitivity analysis, severe ILD was defined as a diagnosis of ILD and administration of intravenous methylprednisolone ≥ 500 mg/day within the period from the first afatinib administration to 1 month after the final administration. This stricter definition was intended to prioritize specificity by focusing on clinically severe ILD cases requiring intensive corticosteroid therapy.

### Statistical and machine learning-based analyses

For patients who developed severe ILD, we investigated the period from the date of the first afatinib administration to the incidence of severe ILD and mortality within 30 days of the incidence of severe ILD. These outcomes were assessed using both the primary and stricter outcome definitions to assess consistency across outcome definitions.

In the field of machine learning, to assess a developed prediction model, the target data are randomly divided into training, validation, and test datasets [19, 20]. Considering that not many patients develop severe ILD, we randomly divided all data into training and test datasets. We developed a prediction model using the training dataset and evaluated the performance of the developed model based on the test dataset. In addition, because the number of severe ILD events is small, separate training and test datasets were constructed for the primary and sensitivity analyses to ensure similar outcome proportions between the training and test datasets within each analysis. Karaman et al. [21] designed an approach using 15% test data and 85% training data, 20% test data and 80% training data, 25% test data and 75% training data, and 30% test data and 70% training data. As the population with a history of ILD is small, we set the ratio of training data high: 85% training data and 15% test data.

Multivariate logistic regression analysis has traditionally been widely employed in clinical research; however, the number of patients who developed severe ILD may be small and the number of variables that can be included in the analysis may be limited [22]. In addition, when the confidence intervals (CIs) of the odds ratios of two variables with significant differences overlap, ranking their variables becomes difficult. In contrast, the Classification and Regression Trees (CART) algorithm developed by Breiman et al. [23] is widely used for outcome prediction in various fields. For example, in clinical medicine, CART is used for predicting heart disease [24] and identifying early lung adenocarcinoma [25].

Using CART, prediction models, such as a logistic regression model, can be developed based on the tree structure. The tree structure represents classification rules, and the tree is known as a “decision tree”. When using CART to develop a model, we can rank the important explanatory variables for the prediction of outcomes and determine the cutoff values of important variables. Therefore, we can easily identify important explanatory variables and interpret them using the developed model. In addition, using the CART model, we can assess the effect of each explanatory variable on the target outcome without considering the relationship between the number of variables and observations. The cutoff values used in the CART models are not predefined but were automatically determined by the CART algorithm in a data-driven manner based on the training dataset. These split points represent values that optimally separated the occurrence of severe ILD within the dataset.

The CART model was used to evaluate factors associated with the incidence of severe ILD. To minimize overfitting, given the small number of severe ILD events, the complexity of the CART model was deliberately constrained by restricting the maximum tree depth to two. This restriction was implemented to limit model flexibility, reduce variance, and enhance interpretability, consistent with the exploratory and hypothesis-generating objectives of the present study. CART analyses were performed using both the primary and stricter outcome definitions to assess the consistency of identified patterns. Although internal validation strategies were considered, the limited number of events imposed substantial constraints; thus, model complexity was restricted, and the CART analysis was intended primarily for exploratory, hypothesis-generating purposes.

Factors associated with the incidence of severe ILD in patients with a history of ILD have not yet been identified. Therefore, we used the following independent variables, including variables commonly considered relevant to ILD based on prior studies: sex [26]; age [26, 27]; body mass index; and history of smoking [27, 28] (Brinkman index), chronic obstructive pulmonary disease [28], and cytotoxic anticancer drug, EGFR-TKI, and immune checkpoint inhibitor use. The designated cancer hospital was also set as an independent variable for facility background.

To evaluate the classification success, receiver operating characteristic (ROC) curve analysis was conducted. In addition, to assess the classification performance, area under the ROC curve (AUC) was calculated. The confidence intervals (CIs) for the AUC values with lower and upper bounds at 95% were calculated.

Results with p-value < 0.05 were considered statistically significant. Stata version 17.0 (Stata Corp., College Station, TX, USA) was used for the statistical analyses and data cleaning. R (version 4.3.1) [29] and the packages “rpart” [30] and “pROC” [31] were used for statistical analyses.

### Ethics approval

We used only an anonymized administrative claims database. This study was confirmed by the ethics committee of Kyoto Pharmaceutical University as it did not require an ethical review, on July 10, 2024 (NR-00001).

## Results

### Patient characteristics

**Fig. 1:**
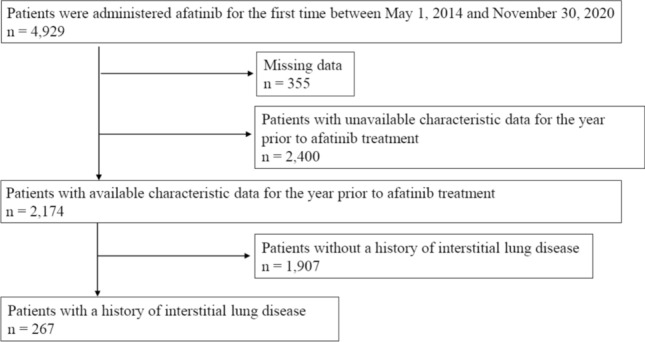
Patient selection flowchart

Figure 1 presents the flowchart of patient selection. A total of 4,929 patients were administered afatinib for the first time between May 1, 2014 and November 30, 2020. Patients with missing background information (355 patients), patients with unavailable patient characteristic data for the year prior to afatinib treatment (2,400 patients), and those without a history of ILD (1,907 patients) were excluded. The final analytic cohort comprising 267 patients with a history of ILD was used for all subsequent analyses. Table 1 presents the characteristics of these patients.

**Table 1:** Patient characteristics

Characteristic	N = 267	
Sex^a^		
Male	108	(40.4)
Female	159	(59.6)
Age (years)^b^	71	[65–76]
Body mass index (kg/m^2^)^a^		
< 18.5	44	(16.5)
18.5–25.0	165	(61.8)
> 25.0	58	(21.7)
Brinkman Index^b^	0	[0–417.5]
History of cytotoxic anticancer drug use^a^	144	(53.9)
History of EGFR-TKI use^a^	144	(53.9)
History of immune checkpoint inhibitor use^a^	22	(8.2)
COPD history^a^	33	(12.4)
Designated cancer hospital^a^	246	(92.1)

^a^n (%), ^b^median [interquartile range]EGFR-TKI, epidermal growth factor receptor tyrosine kinase inhibitor; COPD, chronic obstructive pulmonary disease

Of the 267 patients, 16 (6.0%) met the primary definition of severe ILD, whereas 251 (94.0%) did not. Among the 16 patients who developed severe ILD, the median [interquartile range] duration from the date of first afatinib administration to the incidence of severe ILD was 66.5 [25.0–127.5] days. The mortality rate within 30 days of severe ILD incidence was 18.8% (3/16).

In the sensitivity analysis using the stricter definition of severe ILD, 9 patients (3.4%) met the definition. The median time from first afatinib administration to severe ILD onset in this subset was 50.0 [26.0–124.0] days. The 30-day mortality rate following severe ILD onset was 33.3% (3/9).

### Evaluation of factors associated with severe ILD incidence

**Fig. 2:**
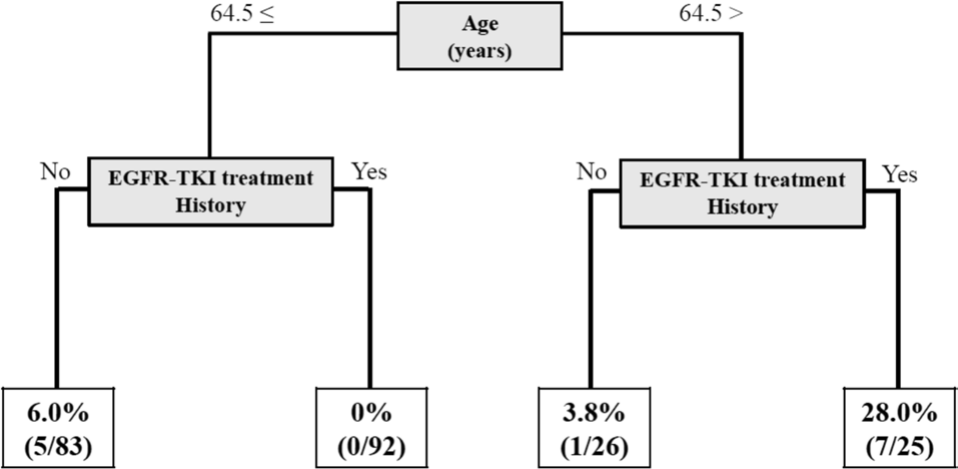
Decision tree for the training dataset developed using CART. CART: Classification and Regression Trees, EGFR-TKI: epidermal growth factor receptor tyrosine kinase inhibitor

**Fig. 3:**
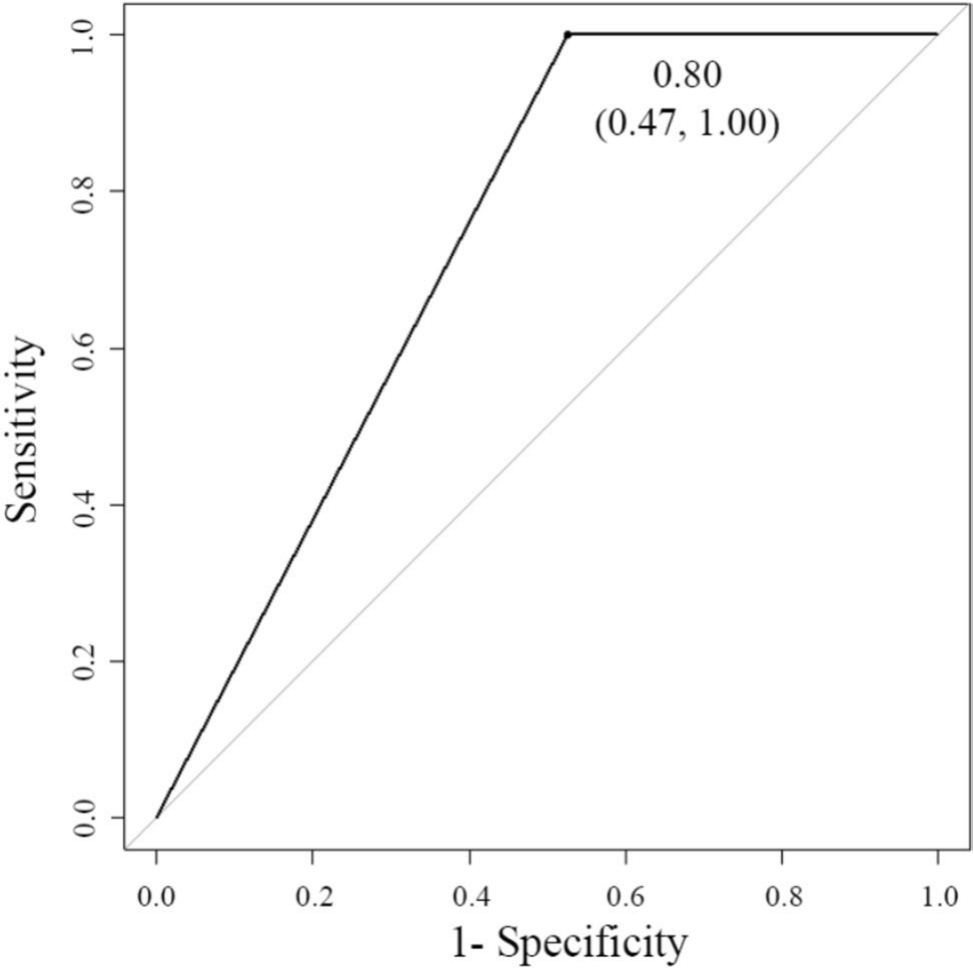
Sensitivity and specificity of the CART model predicting severe ILD development using the test dataset. CART: Classification and Regression Trees, ILD: interstitial lung disease

We randomly divided the original dataset (n = 267) into test (n = 41) and training (n = 226) datasets. In the primary analysis, a decision tree for the training data based on the CART model is shown in Fig. 2. Patients aged < 64.5 years with an EGFR-TKI treatment history were more likely to develop severe ILD (7/25, 28.0%). For the CART model predicting the development of severe ILD withthe test dataset, the AUC was 0.74 (95% CI 0.66–0.82), sensitivity was 1.00, and specificity was 0.47 (Fig. 3).

**Fig. 4:**
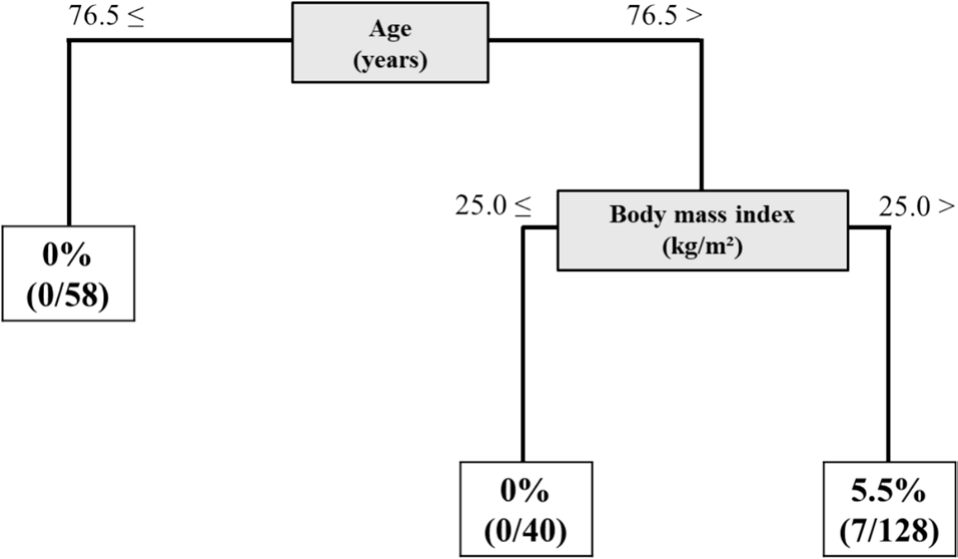
Decision tree for the training dataset developed using CART (sensitivity analysis). CART: Classification and Regression Trees

**Fig. 5:**
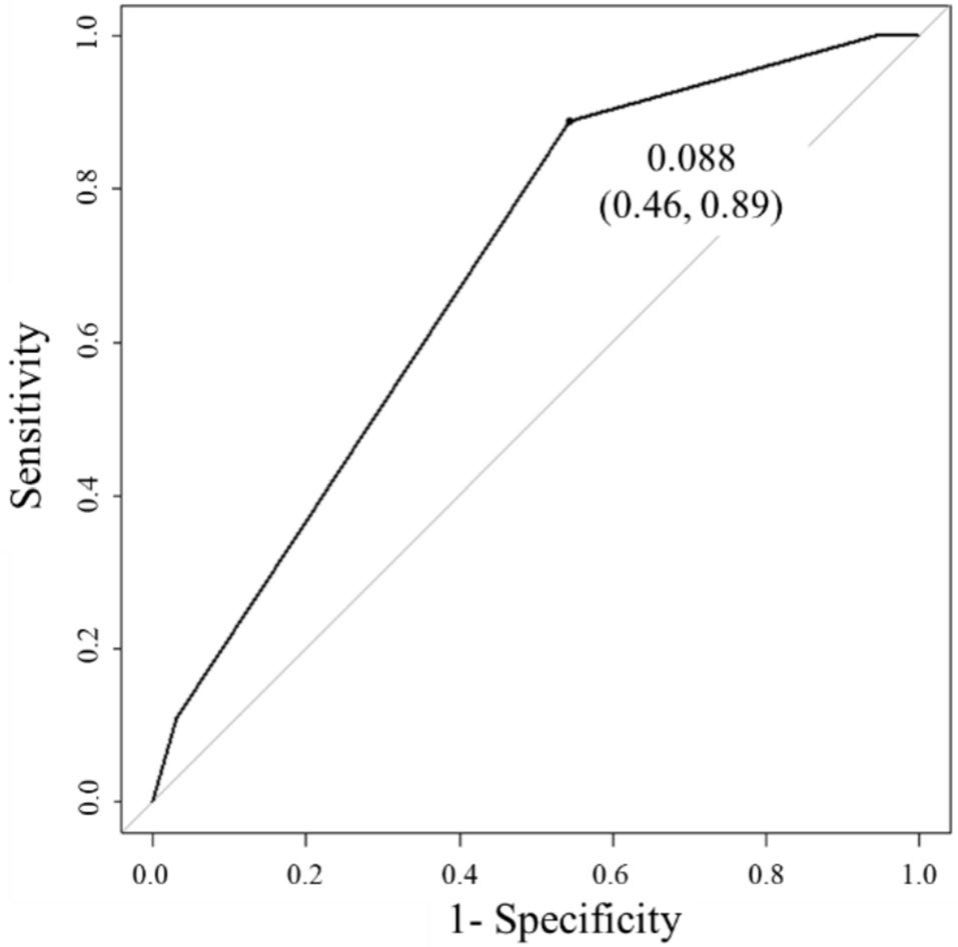
Sensitivity and specificity of the CART model predicting severe ILD development using the test dataset (sensitivity analysis). CART: Classification and Regression Trees, ILD: interstitial lung disease

In the sensitivity analysis using the stricter severe ILD definition, the CART-based decision tree revealed that patients aged < 76.5 years with a body mass index < 25.0 kg/m^2^ had a higher incidence of severe ILD (7/128, 5.5%) (Fig. 4). In the test dataset, the model achieved an AUC of 0.69 (95% CI 0.60–0.78), sensitivity of 0.89, and specificity of 0.46 (Fig. 5).

## Discussion

To our knowledge, no study has evaluated the safety of afatinib in patients with lung cancer and a history of ILD using data from a large cohort. In the present study, 12.3% (267/2,174) of patients who received afatinib had a history of ILD, indicating that afatinib is administered to such patients in routine practice despite safety concerns. In the primary analysis, severe ILD occurred in 6.0% (16/267) of patients with a history of ILD who initiated afatinib. Because outcome misclassification is a recognized limitation of claims-based research, we performed a sensitivity analysis using a stricter operational definition of severe ILD. Under the stricter definition, the number of events decreased from 16 to 9 (3.4%, 9/267), and all 9 cases identified using the stricter definition were included among the 16 cases identified in the primary analysis. This pattern suggests that the stricter definition likely identified a subset of cases with higher clinical certainty or greater severity.

In a randomized controlled trial that excluded patients with a history of ILD, the incidence of severe ILD associated with afatinib administration was 0.4% (1/239) [9]. In contrast, in the present study, the incidence was 6.0% in the primary analysis and 3.4% in the sensitivity analysis. These estimates suggest a similarly range to those reported in a previous study focusing on patients with a history of ILD (6.9%) [15]. Although the absolute incidence of severe ILD depends on the operational outcome definition used and should therefore be interpreted cautiously, the overall pattern supports the clinical concern that a history of ILD experience severe ILD more frequently after afatinib initiation.

Mortality within 30 days of the severe ILD incidence was 18.8% (3/16) in the primary analysis and 33.3% (3/9) in the sensitivity analysis. Despite the small number of events, these findings underscore that severe ILD can be a potentially fatal complication. Therefore, identifying patient profiles associated with severe ILD development in patients with a history of ILD would be clinically relevant.

In the primary analysis, in the decision tree for the training dataset developed using CART, patients aged < 64.5 years with an EGFR-TKI treatment history showed a higher incidence of severe ILD (7/25, 28.0%). On the other hand, in the sensitivity analysis using the stricter outcome definition, the CART model produced a different split structure, where patients aged < 76.5 years with a body mass index < 25.0 kg/m^2^ had a higher incidence of severe ILD (7/128, 5.5%). Although the split variables and thresholds differed between the primary and sensitivity analyses, both consistently suggested that severe ILD events were not evenly distributed among patients with a history of ILD but tended to cluster within identifiable subgroups.

Chronic inflammatory conditions of pulmonary tissues are reportedly related to ILD incidence [32]. As cytokine production increases with age [33], similar to the results of a previous study [26], we predicted that older age would be associated with a higher incidence of severe ILD. However, in the present study, CART analyses in both the primary and sensitivity analyses consistently placed the age split on the younger side, indicating that severe ILD events were more concentrated among younger patients with a history of ILD. This finding should be interpreted with caution. As advanced age and history of ILD have been reported to be associated with EGFR-TKI–induced ILD [26, 27], elderly patients with a history of ILD are generally less likely to receive afatinib therapy due to safety concerns. Therefore, elderly patients who received afatinib might represent a population with a relatively lower incidence of severe ILD, potentially leading to a selection bias. In addition, afatinib dose reduction or adjustment of treatment duration may have been more frequently implemented in elderly patients, which could have contributed to a lower observed incidence of ILD.

Accordingly, the observed age-related split may reflect treatment selection and residual confounding rather than a biological protective effect of older age; however, a true biological association cannot be excluded. Furthermore, the primary objective of the CART analyses was not to establish definitive clinical decision rules, but to explore whether the risk of severe ILD among patients with a history of ILD is uniformly distributed or concentrated within specific patient profiles. Given that the biological mechanisms underlying EGFR-TKI–induced ILD remain poorly understood, the identified split variables and cutoff values, including the age-related pattern, should be interpreted as exploratory, hypothesis-generating findings that warrant further external validation.

For the CART models predicting the development of severe ILD using the test datasets, the primary analysis showed an AUC of 0.74 (95% CI 0.66–0.82), with a sensitivity of 1.00 and a specificity of 0.47, whereas the sensitivity analysis using the stricter definition yielded an AUC of 0.69 (95% CI 0.60–0.78), a sensitivity of 0.89, and a specificity of 0.46. Taken together, these results indicate moderate discriminative ability; however, given the extremely small number of events in the test datasets, these performance estimates (including AUC, sensitivity, and specificity) may be unstable. In particular, the observed high sensitivity—especially the perfect sensitivity in the primary analysis—should be interpreted with caution and should not be viewed as reliable predictive performance. From a clinical perspective, prioritizing sensitivity may be advantageous for a risk-alert purpose, because failure to identify potentially high-risk patients could result in serious clinical consequences. Accordingly, the CART models should not be regarded as definitive rules for determining afatinib eligibility, but rather as exploratory risk alert tools designed to flag patients who may require careful clinical assessment and closer monitoring. This emphasis on sensitivity inevitably comes at the expense of specificity, resulting in a higher false-positive rate; however, such a trade-off may be acceptable in this context given the potentially life-threatening nature of severe ILD and the serious clinical consequences of missed cases.

A major strength of this study is that the CART model enabled us to not only rank variables associated with the outcome but also consider variables that could not be included in the multivariate logistic regression analysis. However, this study has certain limitations. First, because this study was based on an administrative claims database, several limitations inherent to this data source should be considered. The administrative claims database used in this study does not cover hospitals throughout Japan, and the study population was limited to selected hospitals. In addition, a substantial proportion of initially identified patients were excluded during cohort construction. These factors may introduce selection effects and limit the generalizability of our findings to broader clinical populations. Information on disease severity and laboratory test results was not available. Therefore, the claims-based operational definition of severe ILD may have resulted in both under-detection of clinically severe cases not treated with high-dose corticosteroids and over-detection of cases treated with corticosteroids for conditions other than severe ILD. Important clinical confounders, including *EGFR* mutation subtype, ILD severity and subtype, and reasons for prior EGFR-TKI discontinuation, were not available in the database. Although afatinib dosing information was available, the clinical context and reasons underlying dose selection and dose reductions could not be fully ascertained. These unmeasured factors may have influenced treatment selection and the incidence of severe ILD in complex ways, potentially biasing observed associations. Furthermore, the inability to distinguish baseline ILD from prior drug-induced ILD represents a major limitation, as these conditions may differ in susceptibility to subsequent severe ILD. This classification may have introduced misclassification and heterogeneity, which could bias the estimated incidence and should be considered when interpreting the results. Further review of individual patient timelines suggested that deaths did not appear to cluster immediately after afatinib initiation (data not shown), although their impact cannot be completely excluded. In addition, because of the nature of the claims database, the timing of afatinib discontinuation could not be reliably ascertained. Consequently, competing risks such as mortality and treatment discontinuation could not be explicitly modeled. In addition, outliers in the data might have affected the analysis results. Second, the mechanisms underlying severe ILD development could not be elucidated. Third, because the number of severe ILD events was extremely small, we did not perform analyses using alternative tree depths or resampling-based validation methods. Such approaches should be considered in future studies with larger datasets to more robustly assess the stability of the CART findings. Despite these limitations, our study provides important information regarding afatinib safety.

## Conclusion

In patients with a history of ILD, the incidence of severe ILD following afatinib treatment was 6.0% based on the primary definition combining ILD diagnostic codes and high-dose intravenous corticosteroid administration. The CART-derived findings should be interpreted as exploratory and hypothesis-generating signals suggesting that severe ILD events may cluster within specific patient profiles. Given the limitations of the claims database, substantial cohort exclusions, and restriction to selected hospitals, the findings should be interpreted cautiously and may have limited generalizability. Further studies are warranted to validate these findings in independent real-world datasets with richer clinical detail and to support more precise stratification of severe ILD occurrence.

## Supplementary Information

Below is the link to the electronic supplementary material.

Supplementary file 1. **Online Resource 1.** Drug definition based on the anatomical therapeutic chemical classification of the European Pharmaceutical Marketing Research Association. **Online Resource 2.** Disease definition based on the International Classification of Diseases, 10th edition. **Online Resource 3.** Operational definitions of severe ILD used in the primary and sensitivity analyses.

## Data Availability

In this study, data owned by Medical Data Vision (MDV) are acquired under contract. Therefore, the data are not available to the public. The authors have no special privileges to access the data. If other researchers wish to use the data, they must purchase it from MDV as well as the authors. To contact MDV, please use the following website: https://www.mdv.co.jp/.
