# Supplementary material for: Safety evaluation of afatinib in patients with a history of interstitial lung disease using data from an administrative claims database in Japan
Source: Int J Clin Pharm. 2026 Mar 17;48(4):1381–9. doi: 10.1007/s11096-026-02114-2 (PMC13369027; doi:10.1007/s11096-026-02114-2)
Supplement: Supplementary file 1 — Online Resource 1. Drug definition based on the anatomical therapeutic chemical classification of the European Pharmaceutical Marketing Research Association. Online Resource 2. Disease definition based on the International Classification of Diseases, 10th edition. Online Resource 3. Operational definitions of severe ILD used in the primary and sensitivity analyses. [file 11096_2026_2114_MOESM1_ESM.docx]

**International Journal of Clinical Pharmacy**

**Safety evaluation of afatinib in patients with a history of interstitial lung disease** **using data from an administrative claims database in Japan**

Ryo Inose^a^*, Kuniyoshi Hayashi^b^, Toshiyuki Sakaeda^c^

^a^ Laboratory of Clinical Pharmacoepidemiology, Kyoto Pharmaceutical University, 5 Misasagi-nakauchi-cho, Yamashina-ku, Kyoto 607-8414, Japan

^b^ Faculty of Data Science, Kyoto Women’s University, 35 Kitahiyoshi-cho, Imakumano, Higashiyama-ku, Kyoto 605-8501, Japan

^c^ Laboratory of Pharmacokinetics, Kyoto Pharmaceutical University, 5 Misasagi-nakauchi-cho, Yamashina-ku, Kyoto 607-8414, Japan

***Corresponding author:** Ryo Inose, Ph.D.

Laboratory of Clinical Pharmacoepidemiology, Kyoto Pharmaceutical University, 5 Misasagi-nakauchi-cho, Yamashina-ku, Kyoto 607-8414, Japan

Telephone: +81-75-595-4600, Fax: +81-75-595-4750

E-mail: inose2019@mb.kyoto-phu.ac.jp

Online Resource 1. Drug definition based on the anatomical therapeutic chemical classification of the European Pharmaceutical Marketing Research Association

| **Drug classification or drug name** | **Anatomical therapeutic chemical classification** |
| --- | --- |
| Cytotoxic anticancer drugs | L1A, L1B, L1C, L1D, and L1F |
| EGFR-TKIs | L1H2 |
| Immune checkpoint inhibitors | L1G5 |

EGFR-TKI: epidermal growth factor receptor tyrosine kinase inhibitorOnline Resource 2. Disease definition based on the International Classification of Diseases, 10^th^ edition

| **Disease name** | **ICD-10 code** |
| --- | --- |
| Lung cancer | C33, C34 |
| Interstitial lung disease | J702, J703, J704, J841, J849 |
| Chronic obstructive pulmonary disease | J41, J43, J44 |

ICD-10: International Classification of Diseases, 10^th^ edition

Online Resource 3. Operational definitions of severe ILD used in the primary and sensitivity analyses

| **Component** | **Primary analysis definition** | **Sensitivity analysis definition** |
| --- | --- | --- |
| ILD diagnosis | Presence of an ICD-10 diagnosis code | Presence of an ICD-10 diagnosis code |
| Corticosteroid treatment | Intravenous methylprednisolone ≥500 mg/day and/or intravenous prednisolone ≥10 mg/day | Intravenous methylprednisolone ≥500 mg/day |
| Time window for outcome assessment | From the first administration of afatinib to 1 month after the final administration | From the first administration of afatinib to 1 month after the final administration |
| Additional notes | Broad definition intended to capture clinically treated severe ILD in routine practice | Stricter definition intended to improve outcome specificity |

ILD: interstitial lung disease

ICD-10: International Classification of Diseases, 10^th^ edition
